# Deciphering the macrophage ferroptosis regulatory network: construction of an ulcerative colitis diagnostic model and investigation of the immune microenvironment based on single-cell and transcriptomic data

**DOI:** 10.3389/fimmu.2026.1758082

**Published:** 2026-04-22

**Authors:** Yifang Zhang, Zongchi Chen, Su Zhang, Yirong Chen, Chenhang Xie, Qinghua Xiao, Taiyong Fang

**Affiliations:** 1 Department of Gastroenterology, The Second Affiliated Hospital of Fujian Medical University, Quanzhou, Fujian, China; 2 Department of Rheumatology, The Nanping First Affiliated Hospital of Fujian Medical University, Nanping, Fujian, China; 3 Department of Gastrointestinal Surgery, The Second Affiliated Hospital, Fujian Medical University, Quanzhou, Fujian, China

**Keywords:** Bioinformatics, ferroptosis, machine learning, macrophage, predictive model, ulcerative colitis

## Abstract

**Background:**

Ulcerative colitis (UC) is a chronic inflammatory bowel disease manifested as persistent mucosal inflammation and immune dysregulation. As key regulators of intestinal immune homeostasis, macrophages are pivotal in the amplification of inflammation and tissue repair in UC. However, the molecular mechanisms associated with ferroptosis in macrophages remain unclear.

**Method:**

We integrated single-cell and bulk colonic transcriptomes to map ferroptosis-associated programs in UC macrophages, prioritized candidate regulators using network/ML frameworks, and performed targeted experimental validation.

**Results:**

We identified a ferroptosis-associated transcriptional program enriched in UC macrophages and derived a four-gene signature (HIF1A, S100A8, CYBB, GLS) linked to redox/iron stress. Among them, CYBB encodes NOX2 (the catalytic subunit of NADPH oxidase), and emerged as a key node associated with enhanced oxidative/iron stress and coordinated changes in ferroptosis-related markers in macrophages.

**Conclusion:**

This study systematically delineates the ferroptosis-associated transcriptional network in macrophages of UC, providing a systems-level framework linking macrophage redox/iron imbalance to ferroptosis vulnerability in UC and nominating CYBB-centered pathways for mechanistic interrogation and stratification-oriented biomarker development, thereby offering novel theoretical insights for immune metabolism mechanisms and targeted therapies in UC.

## 1 Introduction

Inflammatory bowel disease refers to a group of chronic nonspecific inflammatory diseases of the intestine, primarily including Crohn’s disease (CD) and ulcerative colitis (UC) (1). The core clinical feature of UC is bloody diarrhea (2). The pathogenesis of UC remains inadequately ascertained and may involve genetic susceptibility, mucosal immune dysregulation, and alterations in the microbiota (3). Recently, the global prevalence of UC has continued to rise, leading to an ever-increasing economic burden. Current treatments include corticosteroids, 5-aminosalicylic acid, biologic agents, and small-molecule inhibitors (4). However, these therapies target limited inflammatory pathways and have failed to fundamentally regulate the dysfunction of immune cells. Therefore, it’s necessary to ascertain new potential therapeutic targets.

As the ‘gatekeepers’ of intestinal immunity and tissue homeostasis, macrophages are abundant in the intestinal mucosa and are actively involved in maintaining the stability of the lamina propria, protecting the intestine from inflammatory damage. Studies have exhibited that macrophages are pivotal in the development of UC, and an imbalance of polarization (enhanced M1-type and reduced M2-type) is a key mechanism contributing to chronic inflammation (5). Additionally, metabolic reprogramming, epigenetic regulation, and interactions with the gut microbiota are considered key factors in the functional changes of macrophages. Studies have revealed that during the progression of UC, tissue-resident macrophages undergo a ‘macrophage disappearance reaction’ (MDR), while inflammatory or infiltrative macrophages markedly expand. This replacement of subpopulations is accompanied by a pronounced rise in the levels of reactive oxygen species (ROS), and resident macrophages are more vulnerable to oxidative stress (OS) (6). Ferroptosis is a regulated iron-dependent cell death characterized by lethal accumulation of lipid peroxides due to intracellular iron overload. It induces the disruption of cell membrane structures and metabolic necrosis (7). Recently, growing evidence denotes that ferroptosis is crucial in the pathogenesis of various intestinal disorders. Specifically, damage of epithelial barrier in UC is strongly linked to the inflammatory response (7, 8).

Notably, the imbalance of iron metabolism in macrophages and enhanced OS contribute to atherosclerosis, diabetic retinopathy, breast cancer, and other diseases (9–11). Also, they are implicated in the amplification of inflammation and tissue damage in UC. However, the ferroptosis-associated molecular networks and their regulatory mechanisms across distinct macrophage subpopulations remain incompletely elucidated, offering new insights for exploring the immunometabolic basis of UC.

This research integrated and analyzed single-cell RNA sequencing data (scRNA-seq) and Bulk RNA sequencing data to map the single-cell transcriptomic landscape of macrophages in UC and normal tissues. Key modules in UC were constructed utilizing high-dimensional weighted gene co-expression network analysis (hdWGCNA) and then intersected with ferroptosis gene sets to identify potential regulatory nodes. Subsequently, machine learning (ML) methods were leveraged to ascertain four hub genes strongly linked to ferroptosis in macrophages (HIF1A, S100A8, CYBB, and GLS). Among them, CYBB encodes NOX2 (the catalytic subunit of NADPH oxidase), which is pivotal in regulating the generation of ROS and lipid peroxidation (12). Through further *in vitro and in vivo* experimental validation, this research revealed the crucial role of the CYBB–ROS–ferroptosis pathway in the functional imbalance of macrophages in UC, providing novel theoretical insights for its molecular mechanisms and potential targeted interventions.

## 2 Materials and methods

### 2.1 Data download

All research data were attained from the Gene Expression Omnibus (GEO) database (http://www.ncbi.nlm.nih.gov/geo/) (13). Specifically, the scRNA-seq data for UC were sourced from GSE182270, encompassing 4 normal samples and 5 UC samples. Datasets related to UC were procured from the GEO database by searching for the keyword ulcerative colitis. The selection criteria were outlined below: (i) The species were Homo sapiens; (ii) All specimens were colonic mucosal tissue; (iii) The specimens included UC colonic mucosal tissue and normal control colonic mucosal tissue (NC). Finally, we selected GSE87473 (21 NC and 106 UC) to develop the model and GSE92415 (21 NC and 162 UC) as the validation set for the model (14, 15). Their microarray platforms were all GPL13158. The bulk transcriptomic expression matrices (GSE87473 and GSE92415; GPL13158) were downloaded from GEO as author-provided processed/normalized (log2-scale) expression values, and all downstream analyses were performed on these normalized matrices.

### 2.2 ScRNA-seq data processing

Seurat R package (V.5.3.0) was leveraged to analyze the scRNA-seq data. First, quality control was performed by retaining cells with a mitochondrial transcript percentage < 25% and 500–5,000 detected genes (nFeature_RNA). These thresholds were chosen in line with commonly used Seurat-based QC practices and were supported by inspection of dataset-specific distributions (mitochondrial percentage and nFeature_RNA), to exclude low-quality/dying cells (high mitochondrial fraction) and potential doublets (extremely high gene counts) while preserving high-quality macrophage profiles. The ScRNA-seq data were then normalized utilizing the NormalizeData function. HVGs were identified using FindVariableFeatures with the vst method (nfeatures = 1,000), which ranks genes by standardized variance after accounting for the mean–variance relationship. The resulting top 1,000 HVGs were used for PCA and downstream dimensionality reduction. Batch effects were corrected utilizing the Harmony R package (V.1.2.3). Next, cells were clustered utilizing the FindNeighbors function and FindClusters function, and clustering results were visualized with tSNE. The FindAllMarkers function was leveraged to ascertain marker genes for every cluster and visualize them. Major cell clusters were annotated utilizing known cell marker genes, as listed in **Supplementary Table 1**.

We extracted the macrophage subset, re-normalized and re-dimensionalized the data, and visualized the clustering results with UMAP. Subsequently, the macrophage subclusters were annotated based upon the differential expression between the UC group (UC-M) and the Normal group (N-M). Differential expression analysis of macrophages between the UC-M and the N-M was implemented utilizing the FindMarkers function with thresholds of |log2FC| = 0.15 and adjusted P = 0.05.

### 2.3 Enrichment analysis

Enrichment analysis of differentially expressed genes (DEGs) in macrophages from the UC-M and N-M was implemented utilizing the clusterProfiler R package (V4.16.0), including gene ontology (GO) analysis, Kyoto encyclopedia of genes and genomes (KEGG) analysis, and gene set enrichment analysis (GSEA).

### 2.4 Pseudotime analysis and intercellular communication analysis

Based upon the preprocessed scRNA-seq data, single-cell trajectory analysis was implemented utilizing the Monocle R package (V2.36.0) to reconstruct the dynamic transitions of macrophage states and infer pseudotime. Intercellular communication networks between UC-M and N-M and other cell populations were inferred and characterized utilizing the CellChat R package (V1.6.1) (16).

### 2.5 Acquisition of ferroptosis-related genes

The FerrDb V2 database (http://www.zhounan.org/ferrdb/current/) was a comprehensive resource for FRGs (17). This research downloaded 835 FRGs from this website (**Supplementary Table 2**).

### 2.6 hdWGCNA

The hdWGCNA R package (V0.4.05) was leveraged to ascertain potential macrophage-related genes linked to UC (18). Macrophages were subsetted from the integrated scRNA-seq object, and network construction was performed using the macrophage scRNA-seq expression matrix rather than bulk RNA-seq data. During network construction and module identification, the optimal soft threshold was selected using TestSoftPowers to build signed networks. The soft-thresholding power (β) was determined following the parsimony principle by selecting the smallest β that achieved a scale-free topology model fit index (R²) ≥ 0.90 (and reached a stable plateau) while maintaining reasonable network connectivity. Based on the scale-free topology fit and connectivity curves (**Figure 2A**), β = 12 was selected. The ConstructNetwork was leveraged to calculate the topological overlap matrix (TOM) and performed hierarchical clustering for module detection (merging threshold = 0.25, minimum module size = 200 genes). We set minModuleSize = 200 as a conservative cutoff to reduce module fragmentation and to avoid overly small, potentially unstable modules, which is particularly important for scRNA-seq–based networks with higher sparsity and technical variability. CIBERSORT For module analysis, ModuleEigengenes was leveraged to calculate module eigengenes, ModuleConnectivity was leveraged to ascertain hub genes, and ModuleFeaturePlot was leveraged to visualize module expression patterns on UMAP. Additionally, FindAllDMEs was leveraged to screen for differentially expressed modules, while ModuleTraitCorrelation was leveraged to analyze associations between module and phenotype. Finally, target module genes were extracted for further analysis. For modules obtained through hdWGCNA, we selected those highly expressed in the UC group and intersected them with DEGs and FRGs (hdWGCNA-DEGs-FRGs).

### 2.7 ML method

To further screen hub genes, four ML methods were employed: Random Forest (RF), SVM-Recursive Feature Elimination (SVM-RFE), Boruta, and XGBoost. Internal model selection was performed using k-fold cross-validation and repeated resampling in the discovery cohort (GSE87473), followed by external validation in an independent cohort (GSE92415). The XGBoost model quantified the contribution of each gene to the model’s predictive performance using feature-importance scores. Utilizing xgboost (V1.7.10.1), importance metrics for all genes were extracted, and the top 8 genes were ascertained as UC-associated key genes. Subsequently, the randomForest (v4.7-1.2) and caret (v7.0-1) packages were employed. SVM-RFE was conducted with 10-fold cross-validation (caret, method = ‘cv’, number = 10) to determine the optimal number of features based on the minimum cross-validated error. The feature importance score for each gene was ascertained utilizing the RF model, with the threshold of Mean Decrease Gini score set to 2 for gene selection. For SVM-RFE, parameters were configured as method = ‘svmRadial’. The number of genes was ascertained based upon the lowest error. Feature selection was performed using Boruta (v8.0.0). For model tuning and internal validation, a repeated 10-fold cross-validation scheme was applied (caret: method = ‘repeatedcv’, number = 10, repeats = 5), and the top-ranked genes were retained. Finally, feature genes identified by all four ML methods were intersected. The pROC R package (V1.0-11) was leveraged to plot ROC curves and evaluate the predictive performance of each model. An area under the curve (AUC) close to 1 indicated strong predictive performance of the model. Model generalizability was assessed by external validation using the independent GSE92415 cohort.

### 2.8 Nomogram

Based upon the four hub genes selected by the four ML methods, a nomogram was developed utilizing the rms R package (V8.0) to assess the risk of disease occurrence. Calibration curves were utilized to appraise the accuracy of the nomogram predictions. Decision curves were leveraged to estimate the net clinical benefit of the model developed utilizing the four hub genes.

### 2.9 Immune infiltration analysis

Immune infiltration analysis was performed using the CIBERSORT R package (V0.1.0) with the LM22 signature matrix in relative mode (default; absolute mode was not enabled), which outputs normalized immune-cell fractions per sample. CIBERSORT is a deconvolution algorithm based on linear support vector regression that infers immune-cell fractions from bulk expression profiles using predefined immune signature genes. CIBERSORT was run with 1,000 permutations (perm = 1000) and quantile normalization disabled (QN = FALSE), and only samples with deconvolution P-values < 0.05 were retained for downstream comparisons; box plots were then used to compare the fractions of 22 immune cell types between UC and NC samples. Spearman’s rank correlation analysis was performed (using the filtered samples) to quantify associations between hub-gene expression and immune cell infiltration (ICI), and correlation heatmaps were used to visualize these relationships.

### 2.10 Statistical analysis

All analyses were implemented utilizing R software (version 4.5.0) and GraphPad Prism (version 10.1.2). For comparisons between two groups, the Wilcoxon signed-rank test was leveraged. Spearman’s rank correlation test was utilized to examine associations between hub genes and ICI. P < 0.05 signified statistical significance.

### 2.11 Cell culture and treatment

Human monocytic leukemia cells (THP-1) were purchased from Wuhan Procell Life Technology Co., Ltd. and cultured in RPMI-1640 (MA0215, MeilunBio) supplemented with 10% fetal bovine serum (FBS, 164210, Procell), penicillin-streptomycin (P1400, Solarbio), and 0.05 mM β-mercaptoethanol (M6230-100ml, McLin) at 37 °C with 5% CO_2_. Cells were treated with 10 ng/ml phorbol 12-myristate 13-acetate (PMA; MedChemExpress, Cat. HY-18739) for twenty-four hours to induce differentiation of THP-1 cells into M0 macrophages. Through preliminary experiments, the optimal concentration of puromycin (MA0318, MeilunBio) was ascertained as 2 µg/mL, and a multiplicity of infection (MOI) of 20 exhibited stronger fluorescent signal intensity in cells. Cells were plated in 6-well plates at a density of approximately 2×10^5^ cells per well, and 1 mL of complete medium was added to resuspend the cells. Based upon the MOI = 20 ascertained by preliminary experiments, the corresponding volume of recombinant lentiviral vector solution (RC207544L4V, ORIGENE) was delivered into each well. Polybrene (8 µg/mL) was also added to boost the efficiency of viral infection. After 4–6 hours of infection, 1 mL of complete medium was added to restore the culture volume. Following infection, cells were cultured for an additional 72 hours before subsequent experiments were conducted.

### 2.12 RT-qPCR

RNA extraction kits were purchased from Beyotime Biotechnology (Shanghai, China, R0027). Total RNA was extracted following the manufacturer’s protocol. Reverse transcription reagents were attained from Takara Bio Inc. (Japan). cDNA was synthesized based upon the manufacturer’s instructions. Finally, the target genes were amplified utilizing an ABI PRISM 7500 PCR instrument (Applied Biosystems, United States). The primers utilized in this research are demonstrated in **Supplementary Table 3**.

### 2.13 Detection of oxidative species

ROS (MA0219, Meilun Bio, Dalian, China) and Fe^2+^ (ADS-F-QT027, AIDISHENG, Jiangsu, China) test kits were leveraged to detect ROS and Fe^2+^ levels in cells. The assays were implemented based upon the manufacturer’s instructions.

### 2.14 CCK8

Cells were seeded in 96-well plates at a density of 1×10^4^ cells per well and incubated for twenty-four hours in a cell culture incubator to ensure cell adhesion and stability. Subsequently, the cells were treated with various concentrations of Erastin (0, 1, 2, 5, 10, 20, 50 μM) in different groups, with each group receiving the corresponding concentration of drug solution. After twenty-four hours of treatment, cell viability was appraised utilizing the CCK-8 reagent. The absorbance (OD value) of each well was measured at a wavelength of 450 nm utilizing a microplate reader. Based upon the results of detection, 10 μM of Erastin was ascertained as the concentration for subsequent experiments.

### 2.15 Animal experiment

Twelve male C57BL/6 mice, aged 6–8 weeks (purchased from Shanghai SLAC Laboratory Animal Co., Ltd.), were used in this study and housed under standard experimental conditions prior to the start of the experiment. The animals were maintained in a temperature-controlled environment at 22 ± 2 °C with a 12-hour light/dark cycle, and were allowed free access to food and water. After one week of acclimatization, the mice were randomly classified into the control group (NC, n = 6) and the DSS-induced group (DSS, n = 6). Within the DSS group, 3.5% dextran sulfate sodium (DSS, MB5535, MeilunBio) was added to the drinking water for 7 consecutive days to induce the UC model. On day 8, the animals were euthanized, and relevant tissue samples were collected for further experimental analysis.

### 2.16 Hematoxylin and Eosin staining

Colon tissues were fixed in 4% paraformaldehyde and embedded in paraffin. After sectioning (5 µm), the sections were dehydrated with ethanol and xylene, followed by staining with H&E. Histopathological evaluation was performed microscopically, with scoring based upon the degree of damage and the extent of inflammatory infiltration (19).

### 2.17 Immunofluorescence staining

For immunofluorescence staining, colon tissue slides were deparaffinized utilizing xylene and ethanol. Antigen retrieval was implemented utilizing Tris-EDTA, and blocking was carried out with 5% BSA (B2064, Sigma). Slides were incubated with primary antibody against CYBB (19013-1-AP, Proteintech) overnight at 4 °C. On day 2, secondary antibody incubation was conducted at 37 °C for 1 hour. Antigen retrieval was performed with citrate buffer. Slides were incubated with primary antibody against F480 (70076, CST) overnight at 4 °C. On day 3, nuclei were counterstained with DAPI for 10 minutes at room temperature in the dark.

## 3 Results

### 3.1 Single-cell transcriptomic landscape of macrophages in normal and UC tissues

**Figure 1:**
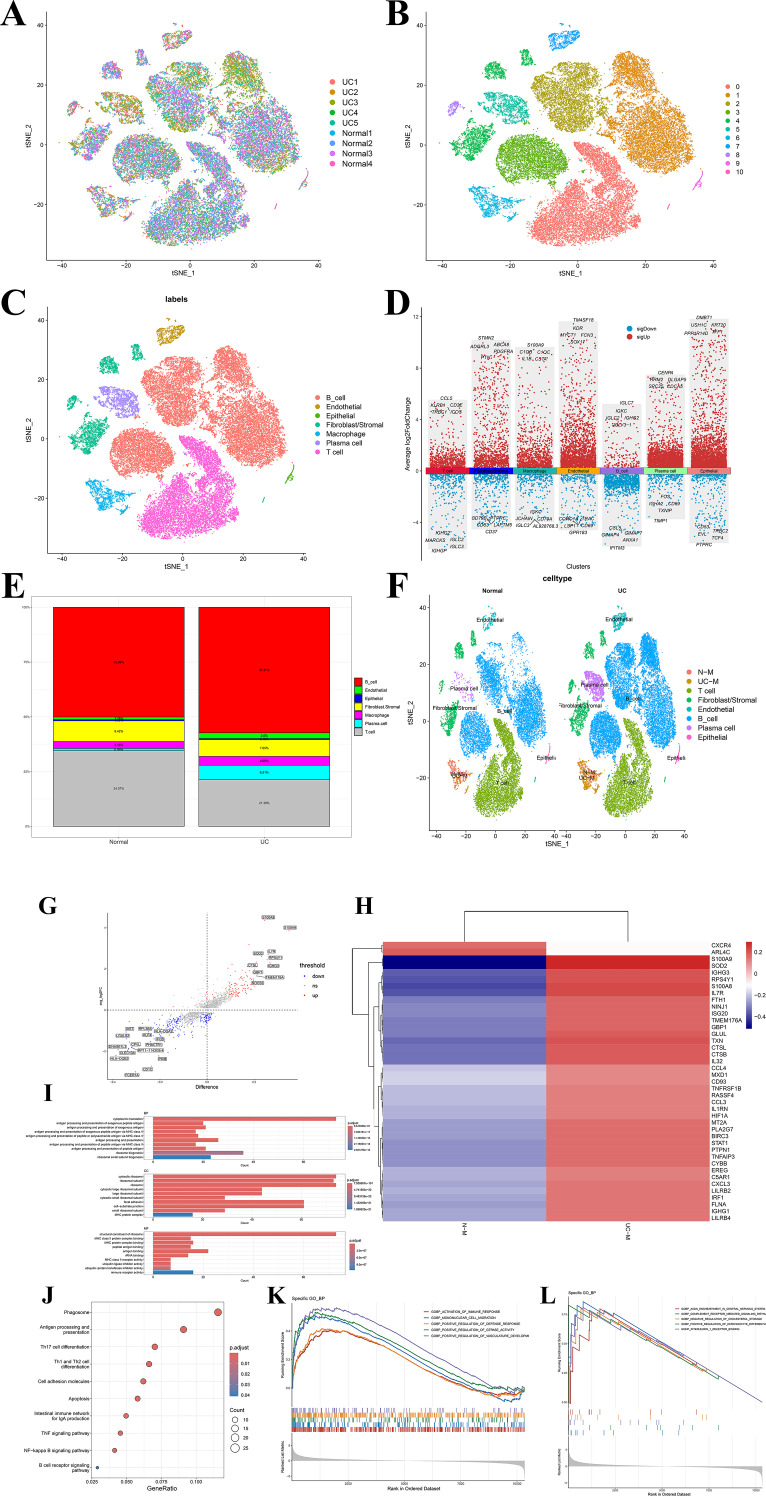
**(A)** Global visualization of single-cell transcriptomes with sample source annotation; **(B)** t-SNE visualization of 11 cell clusters; **(C)** Annotation and spatial distribution of major cell types; **(D)** Visualization of the top 10 logFC-ranked genes in each cell type; **(E)** Differences in cellular composition between normal and UC (ulcerative colitis) tissues; **(F)** Embedded distribution of macrophage subpopulations across different states. After extracting macrophages from all cells, the t-SNE distribution is color-coded by source state (N-M, normal-derived macrophages; UC-M, UC-derived macrophages) to visually demonstrate disease status-related transcriptomic heterogeneity and population displacement; **(G)** Differentially expressed genes (DEGs) in UC-M compared to N-M; **(H)** Heatmap of differentially expressed genes between N-M and UC-M macrophages; **(I)** GO enrichment analysis of differentially expressed genes; **(J)** KEGG pathway enrichment analysis of DEGs between N-M and UC-M macrophages; **(K)** GSEA plots of representative GO terms in UC-M macrophages; **(L)** GSEA plots of additional enriched GO terms related to metabolism and signaling.

To elucidate the potential pathological role of macrophages in UC, we analyzed scRNA-seq data (4 normal control samples and 5 UC samples) from GSE182270. t-SNE dimensionality reduction visualization revealed clear separation between normal and UC samples at the overall transcriptomic level (**Figure 1A**). In total, 11 distinct cell clusters were identified (**Figure 1B**). Based upon typical cell marker genes, major cell populations were identified in the intestinal tissue, encompassing T cells, B cells, plasma cells, epithelial cells, fibroblasts/stromal cells, endothelial cells, and macrophage populations (**Figure 1C**). We next performed differential expression analyses across the major cell populations. Within the macrophage population, upregulated genes included C1QC, C1QB, CSTA, S100A9, and IL1B, while downregulated genes included IGKC, JCHAIN, CD79A, and IGLC3 (**Figure 1D**). Inter-group comparison analysis revealed a pronounced increase in the proportions of macrophages, B cells, and plasma cells in UC tissues (**Figure 1E**). Macrophages were isolated and subjected to standardization and PCA dimensionality reduction. Two distinct macrophage clusters were identified (UC-M and N-M) (**Figure 1F**). Differential expression analysis between these clusters was performed, generating a volcano plot and corresponding heatmap (**Figures 1G, H**). GO enrichment analysis of DEGs further indicated that UC-M macrophages were primarily enriched in pathways linked to antigen processing and presentation (MHC-II dependent), inflammatory cytokine signaling, and immune receptor activity (**Figure 1I**). KEGG pathway enrichment analysis revealed marked enrichment of DEGs in the NF-κB signaling pathway, TNF signaling pathway, and apoptosis pathways (**Figure 1J**). This suggested it was pivotal in the release of inflammatory cytokine and regulation of cell death. Furthermore, significant enrichment of the phagosome and antigen processing and presentation pathways highlighted their functional enhancement in antigen recognition and immune activation. Notably, the results of enrichment also revealed several pathways associated with T/B cell synergistic immune responses, which collectively formed a macrophage-driven immune-inflammatory network in the UC mucosa. This indicated that macrophages may amplify local intestinal inflammation through cooperation with adaptive immune cells. DEGs were primarily enriched in immune response– and inflammation-related signaling pathways. GSEA analysis demonstrated that macrophages were considerably enriched in various inflammatory and immune-related biological processes (**Figure 1K**). Moreover, they exhibited enrichment in typical inflammatory amplification pathways like the complement receptor-mediated signaling pathway and interleukin-1 receptor binding (**Figure 1L**). This further emphasized the key role of macrophages in inflammation signaling, recruitment of immune cells, and amplification of immune response in UC tissues. These findings revealed the macrophages may exert critical effects in inflammation and immune regulation within UC.

### 3.2 hdWGCNA revealed key modules associated with macrophages in UC

**Figure 2:**
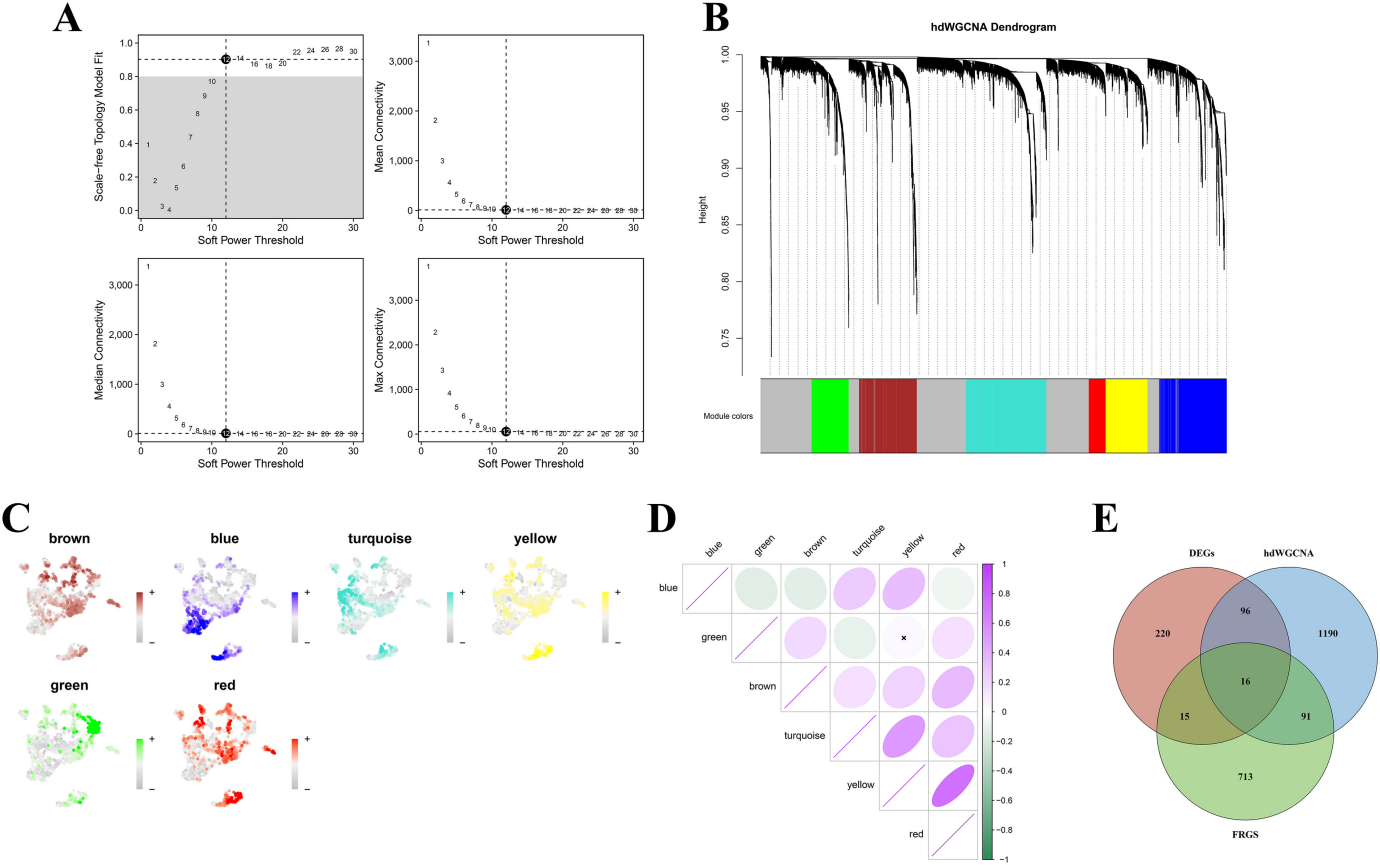
**(A)** Selection of soft‐thresholding power for hdWGCNA (β = 12 was chosen as the smallest value achieving a stable high scale-free topology model fit while maintaining reasonable network connectivity); **(B)** Gene dendrogram and module identification; **(C)** t-SNE plot derived from scRNA-seq data; **(D)** Correlation among module eigengenes; **(E)** Intersection of hdWGCNA modules, DEGs, and ferroptosis-related genes (FRGs) (Venn diagram).

hdWGCNA was constructed based upon the macrophage scRNA-seq transcriptome. A soft threshold of 12 was ascertained to build a scale-free network, which was classified into 6 modules (including blue, green, brown, turquoise, yellow, and red) (**Figures 2A, B**). Notably, the blue and green modules were highly enriched in macrophages derived from UC tissues (**Figure 2C**). The interrelations between the modules were ascertained. A robust negative interrelation was detected between the blue and green modules (**Figure 2D**), while a positive interrelation was detected between the yellow and red modules.

To elucidate the function of these modules in ferroptosis, the FRGs sets were downloaded and the intersection analysis between genes from the blue and green modules, DEGs, and FRGs was implemented. The results revealed a set of key candidate genes that exhibited core module significance, differential expression, and ferroptosis characteristics (**Figure 2E**). These genes potentially contribute to the inflammatory functions of macrophages in UC and might be involved in regulating the sensitivity of ferroptosis.

### 3.3 Pseudotime analysis and cell communication analysis

**Figure 3:**
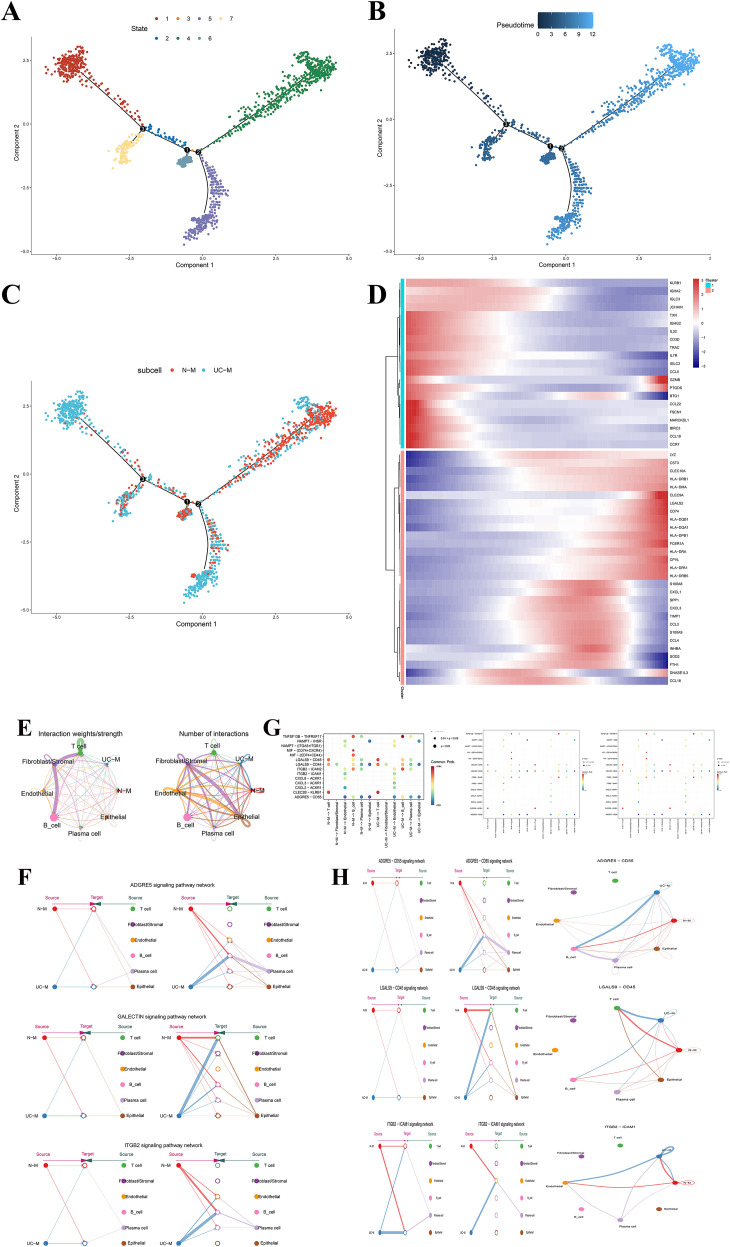
**(A–C)** Developmental trajectory analysis of macrophages; **(D)** Heatmap of representative gene expression along trajectories and state transitions; **(E)** Strength and quantity of intercellular communication between cell populations; **(F)** Communication differences in ADGRE5-CD55, GALECTIN, and ITGB2 signaling axes between N-M and UC-M; **(G)** Network depicting cell-cell ligand-receptor (LR) pairs and cytokine-related pathways illustrates interaction patterns of N-M and UC-M with other cell populations; **(H)** Reconstruction of ADGRE5–CD55, LGALS9–CD45, and ITGB2–ICAM1 signaling networks.

Pseudotime trajectory analysis of macrophages revealed that macrophages were classified into multiple distinct states (state 1-7) along the trajectory (**Figures 3A, B**). Macrophages derived from normal tissues (N-M) were mainly concentrated in state 4 and state 6. Characteristic genes like HLA-DRA, HLA-DRB1, CD74, and CPVL indicated that N-M were involved in antigen presentation and the maintenance of immune homeostasis. In contrast, macrophages derived from UC tissues (UC-M) were more distributed in state 1, state 5, and state 7, showing high expression of genes such as S100A8, S100A9, CXCL1, CCL3, and SPP1. These genes were strongly linked to inflammation amplification, secretion of chemokine, and tissue damage (**Figures 3C, D**). Notably, pseudotime analysis of UC-M revealed a trend toward differentiation into diverse cell fates. Some UC-M exhibited a high-inflammatory phenotype (with high expression of IL1B, CCL4, CXCL3), while others showed enrichment of genes linked to the regulation of ferroptosis (e.g., FTH1 and SOD2). This suggested that macrophages might follow two distinct cell fates within UC: an inflammation-driven phenotype and a ferroptosis-susceptible phenotype.

To further characterize macrophage functions within the UC microenvironment, we inferred intercellular communication using CellChat based on the scRNA-seq data. We observed that UC-M showed a marked increase in the number of inferred communications, with the strongest interactions involving fibroblasts/stromal cells, endothelial cells, and N-M (**Figure 3E**). Notably, UC-M and N-M interacted with other cell types through various ligand-receptor pairs, including ADGRE5-CD55, LGALS9-CD45, and ITGB2-ICAM1 (**Figures 3F–H**).

These results denoted that macrophages within UC did not act independently. They established abnormal communication networks with immune and stromal cells through specific ligand-receptor axes (e.g., ADGRE5-CD55, LGALS9-CD45, ITGB2-ICAM1), thereby amplifying the inflammatory response and exacerbating tissue damage.

### 3.4 ML analysis revealed ferroptosis-related characteristic genes in UC-M

**Figure 4:**
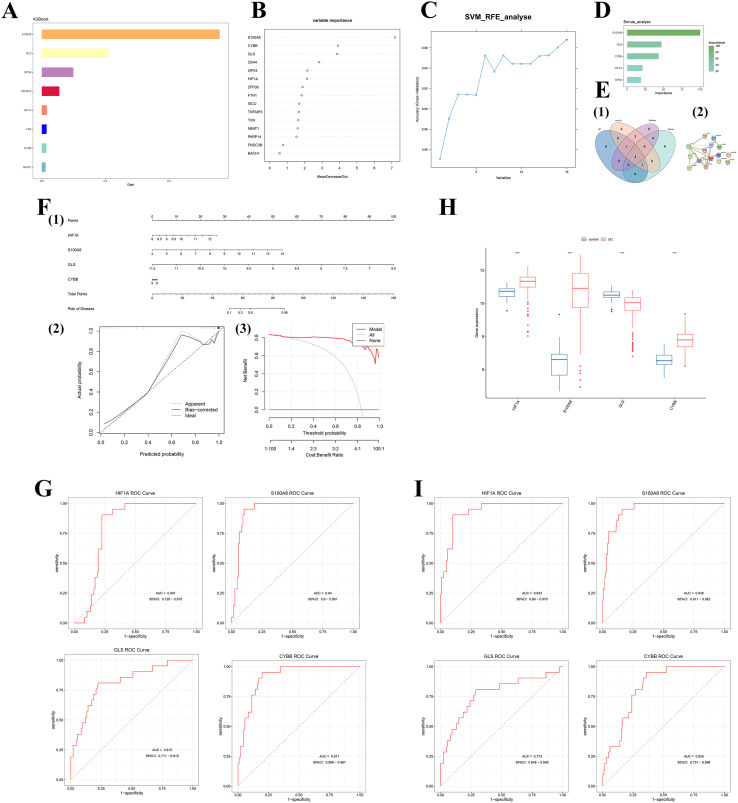
**(A)** Feature importance from XGBoost model; **(B)** Variable importance from Random Forest; **(C)** SVM-RFE with cross-validated accuracy; **(D)** Boruta feature selection; **(E)** Consensus hub genes and protein–protein interaction (PPI) network; **(F)** Clinical utility of the four-gene model: 4F-1: Nomogram constructed based on HIF1A, S100A8, CYBB, and GLS. Individual risk of UC is calculated by summing scores corresponding to gene expression levels. 4F-2: Calibration curve demonstrating high concordance between model-predicted probabilities and actual incidence. 4F-3: Decision curve analysis (DCA); **(G)** Receiver operating characteristic (ROC) analysis for hub genes in the validation cohort; **(H)** Differential expression of four hub genes between normal and UC tissues; **(I)** Box plots showing expression differences of hub genes across disease states (Adjusted based on contextual analysis).

Eight key genes were identified using XGBoost (**Figure 4A**), and RF ranked the top six genes by importance (**Figure 4B**). Additionally, 14 genes were identified through SVM-RFE (**Figure 4C**), and 5 genes were identified through Bruta (**Figure 4D**). Ultimately, 4 hub genes were identified, encompassing HIF1A, S100A8, GLS, and CYBB (**Figure 4E-1**). PPI analysis exhibited that these genes formed a tightly connected interaction module in the network and were strongly linked to multiple ferroptosis regulatory molecules (like GPX4, FTH1, and TXN) (**Figure 4E-2**). In this network, CYBB (NOX2) was centrally located and strongly linked to several core regulators of ferroptosis, suggesting that it might act as an upstream promoter in macrophage ferroptosis by regulating the generation of ROS and accumulation of lipid peroxidation. The nomogram demonstrated that the expression levels of each gene contributed weighted scores to the risk of disease, with higher total scores signifying a greater probability of UC occurrence (**Figure 4F-1**). The calibration curve was leveraged to estimate the stability of the model. Results exhibited that the nomogram had reliable predictive performance (**Figure 4F-2**). Furthermore, Decision curve analysis (DCA) revealed that the model provided significant net clinical benefit across a wide range of threshold probabilities (**Figure 4F-3**). This suggests that the constructed nomogram shows reliable performance in this tissue transcriptomic cohort and may have potential value for research-oriented risk stratification; however, its clinical utility requires prospective validation in independent cohorts. To evaluate the diagnostic potential of these four hub genes, receiver operating characteristic (ROC) curve analysis was implemented. The ROC analysis exhibited that the AUC for all four hub genes was greater than 0.8, indicating good discriminative ability of the four-gene signature in colonic tissue transcriptomic cohorts (**Figure 4G**). Notably, this performance reflects an in silico, tissue-based proof-of-concept and warrants further external validation and assay translation prior to consideration of any potential clinical application. Box plot analysis revealed an increase in the expression of HIF1A, S100A8, and CYBB, with S100A8 exhibiting the most significant upregulation in UC. The expression of GLS was decreased (**Figure 4H**). Group differences in gene expression (UC vs control) were assessed using a two-sided Wilcoxon rank-sum test (Mann–Whitney U test). The GSE92415 dataset was utilized as an external validation cohort. Discriminatory evaluation of the four ferroptosis-related hub genes was implemented. The results exhibited that the AUC for all four hub genes was > 0.77 (**Figure 4I**).

### 3.5 Immune infiltration analysis

**Figure 5:**
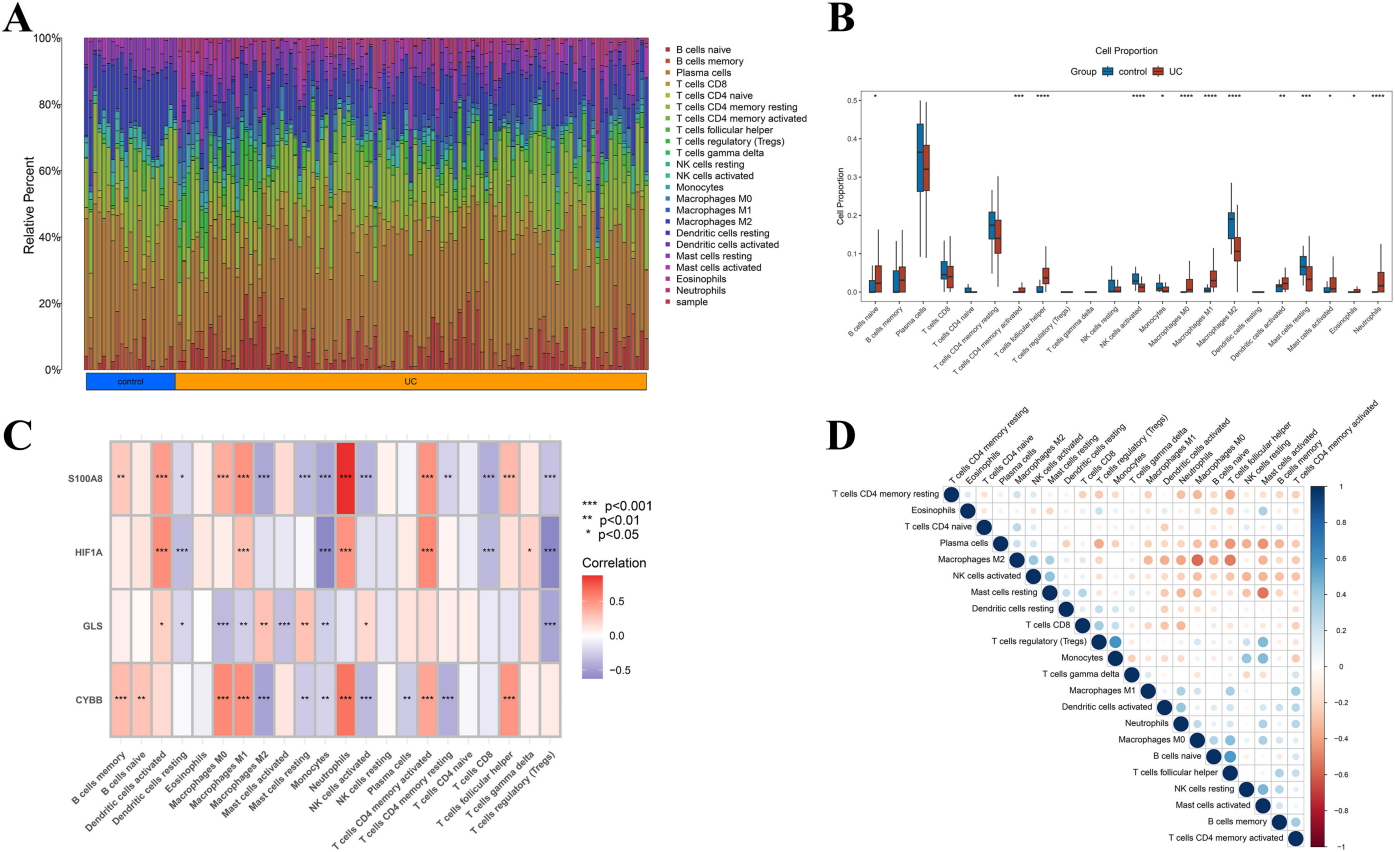
**(A)** Immune infiltration composition in UC and control samples; **(B)** Difference of 22 immune cell types between UC and controls; **(C)** Correlation heatmap between four hub genes and immune infiltration; **(D)** Correlation analysis of immune cell differentials; blue indicates positive correlation, and red indicates negative correlation. These symbols indicate statistical significance levels: *represents p<0.05; ** represents p<0.01; *** represents p<0.001; **** represents p<0.0001.

Through CIBERSORT analysis, the relative abundance of 22 immune cell types in UC and NC samples was quantified (**Figure 5A**). The results exhibited a marked remodeling in the composition of immune cells within UC tissues. The changes in macrophage-related subpopulations were the most prominent (**Figure 5B**). Relative to the control group, M0 and M1 macrophages were considerably elevated in UC (p<0.001), whereas M2 macrophages were considerably decreased (p<0.01). This suggested a marked shift in the polarization of macrophages from the homeostatic phenotype toward the pro-inflammatory phenotype. Additionally, the proportions of neutrophils and activated dendritic cells were elevated, while NK cells were considerably reduced. Correlation analysis further denoted that the hub genes S100A8, HIF1A, and CYBB were considerably positively linked to the infiltration level of M1 macrophages (p<0.001). Meanwhile, GLS exhibited a negative interrelation with M1 macrophages (p<0.01) (**Figure 5C**). Collectively, these results suggested an association between ferroptosis-related hub genes and macrophage infiltration/polarization-related signatures in UC. Notably, we provided functional evidence supporting a role for CYBB in modulating oxidative stress and lipid peroxidation–linked ferroptotic responses, whereas the causal contributions of HIF1A, S100A8, and GLS to macrophage polarization warranted further mechanistic investigation. M0 macrophages were negatively correlated with M2 macrophages and positively correlated with M1 macrophages (**Figure 5D**).

### 3.6 CYBB expression was elevated in colonic macrophages of DSS-induced mice within UC

**Figure 6:**
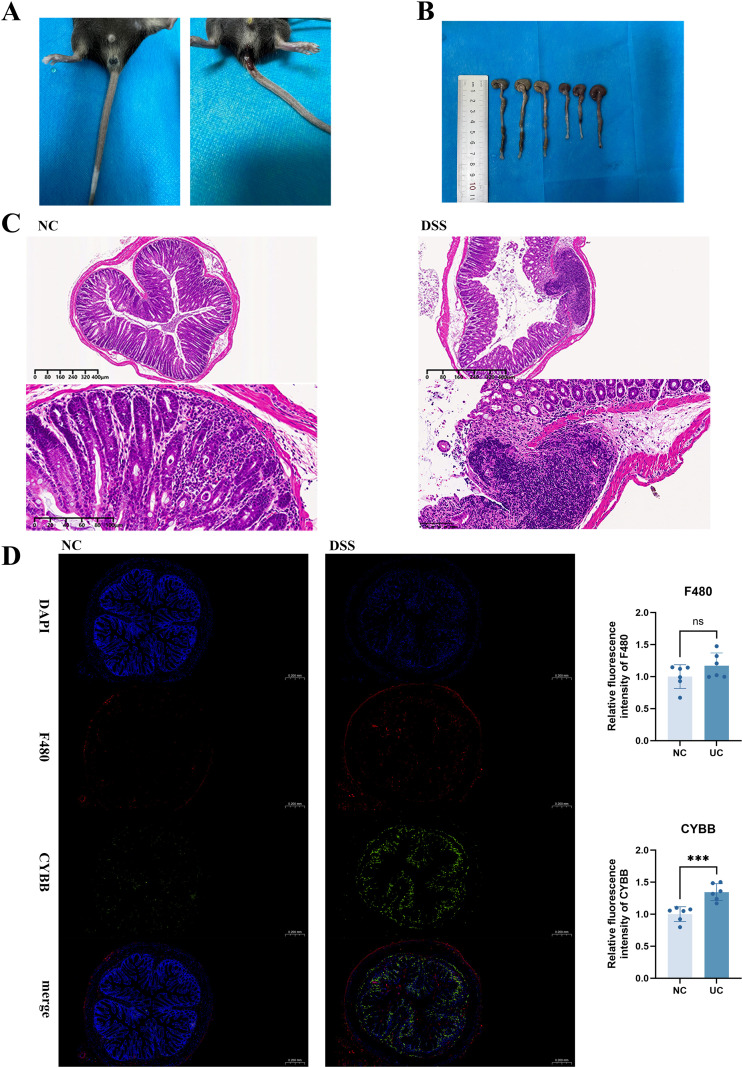
**(A)** Stool characteristics of normal control (NC) and 3.5% DSS-treated mice. DSS group exhibited bloody stools; **(B)** Changes in length of Colon in NC and 3.5% DSS-treated mice; **(C)** H&E staining of mouse colon tissues (Scale: 400 μm, 100 μm); **(D)** Immunofluorescence staining of CYBB and F4/80. Blue: DAPI nuclear stain; Green: CYBB localization; Red: F4/80 localization, *** indicates p<0.001 (highly significant); ns stands for “not significant” (p≥0.05).

A mouse model of UC was established by adding 3.5% DSS to drinking water for seven consecutive days. The mice could freely drink the DSS solution. We observed that, compared with normal mice, DSS-treated mice exhibited body-weight loss, diarrhea, fecal occult blood or overt bleeding, and shortened colons (**Figures 6A, B**). After H&E staining of colon tissues, DSS-treated mice exhibited extensive epithelial erosion, distortion of crypt structure, depletion of goblet cells, and significant infiltration of inflammatory cells in the lamina propria relative to the normal control group. This confirmed the successful development of the UC model (**Figure 6C**).

To visualize the localization of CYBB, immunofluorescence staining was performed on colon sections with DAPI (blue, nuclear marker), F4/80 (red, macrophage marker), and CYBB (green). Within the control group, sparse F4/80^+^ macrophages were distributed along the lamina propria, with weak signals of CYBB fluorescence. In contrast, DSS-treated colon tissue displayed intense green CYBB fluorescence that co-localized with numerous red F4/80^+^ macrophages, particularly in areas with severe epithelial damage (**Figure 6D**). The merged image clearly exhibited yellow and orange overlapping signals, confirming the co-expression of CYBB in infiltrating macrophages. Quantification showed that CYBB fluorescence intensity was significantly increased in DSS-treated mice compared with controls (p<0.001).

These findings suggested that CYBB was considerably upregulated in colonic macrophages of DSS-induced mice within UC, providing *in vivo* evidence that CYBB-driven ROS might participate in macrophage-mediated ferroptotic inflammation and epithelial injury within UC.

### 3.7 CYBB overexpression enhanced ROS and ferroptosis-related responses in macrophages

**Figure 7:**
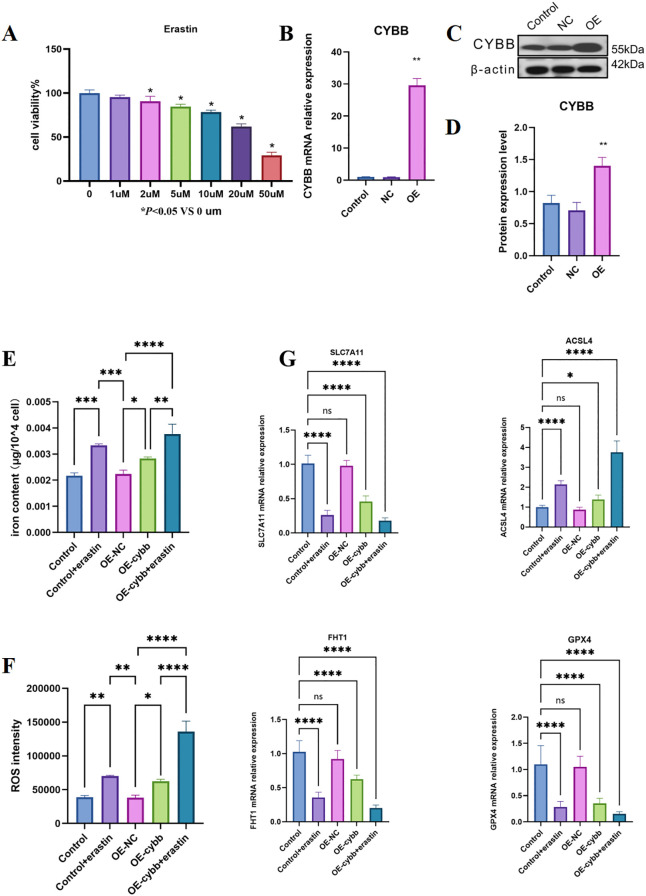
**(A)** Erastin dose-ranging study and determination of treatment concentration; **(B)** Transcriptional validation of CYBB overexpression; **(C, D)** Protein-level validation of CYBB overexpression; **(E)** Content of intracellular ferrous iron (Fe^2+^); **(F)** Intensity of intracellular reactive oxygen species (ROS); **(G)** Transcriptional profile of key molecules in ferroptosis: mRNA levels of ACSL4, SLC7A11/GPX4, and FTH1. Data are presented as mean ± SD from n = 3. Statistical significance was evaluated using one-way ANOVA followed by Tukey’s multiple-comparisons test. ns, not significant; P < 0.05, P < 0.01, P < 0.001, P < 0.0001. These symbols indicate statistical significance levels: *represents p<0.05; ** represents p<0.01; *** represents p<0.001; **** represents p<0.0001.

We first used THP-1 cells as a model and conducted a dose optimization utilizing the CCK8 assay to ascertain the appropriate concentration of 10 µM Erastin for subsequent experiments (**Figure 7A**). After PMA induction of THP-1 cells to obtain M0 macrophages, a macrophage cell line stably over-expressing CYBB was established (**Figures 7B–D**). Results exhibited that, relative to the control and OE-NC groups, the OE-CYBB group exhibited enhanced levels of Fe^2+^ and ROS. Upon stimulation with Erastin, the levels of Fe^2+^ and ROS in the OE-CYBB group further increased (**Figures 7E, F**).

**Figure 8:**
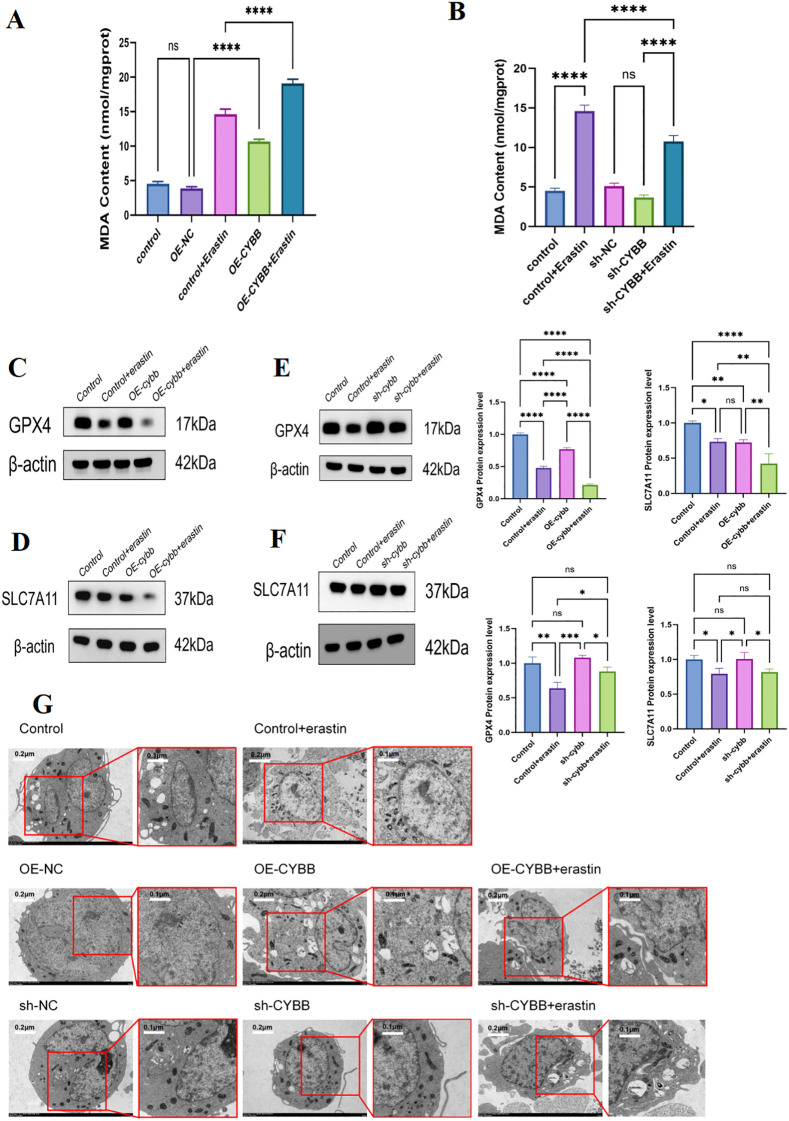
**(A)** MDA levels in OE-NC and OE-CYBB cells with/without erastin. **(B)** MDA levels in sh-NC and sh-CYBB cells with/without erastin. **(C, D)** Representative immunoblots **(C)** and quantification **(D)** of GPX4 and SLC7A11 in OE-NC and OE-CYBB cells ± erastin. **(E, F)** Representative immunoblots **(E)** and quantification **(F)** of GPX4 and SLC7A11 in sh-NC and sh-CYBB cells ± erastin. **(G)** Representative transmission electron microscopy (TEM) images showing mitochondrial ultrastructure in macrophages under the indicated conditions (Control, Control+Erastin, OE-NC, OE-CYBB, OE-CYBB+Erastin, sh-NC, sh-CYBB, and sh-CYBB+Erastin). Red boxes indicate regions enlarged in the corresponding zoomed views. CYBB overexpression exacerbated Erastin-associated ferroptosis-like mitochondrial damage, characterized by mitochondrial shrinkage and cristae disruption, whereas CYBB knockdown partially preserved mitochondrial integrity under Erastin challenge. Scale bar, 2.0 µm. For immunoblot quantification, band intensities were quantified by densitometry and normalized to β-actin, based on n = 3. For MDA quantification, data are presented as mean ± SD from n = 3. Statistical analyses were performed using one-way ANOVA followed by Tukey’s multiple-comparisons test. ns, not significant; *P < 0.05, **P < 0.01, ***P < 0.001, ****P < 0.0001.

PCR analysis detected the levels of key ferroptosis-driving gene (ACSL4) and ferroptosis-protective genes (SLC7A11, GPX4) (**Figure 7G**). Results denoted that the levels of SLC7A11 and GPX4 were downregulated within the OE-CYBB group and further suppressed under the stimulation of Erastin. The ferroptosis-driving gene ACSL4 exhibited an upward trend within the OE-CYBB and OE-CYBB+Erastin groups. Levels of FTH1 decreased in the OE-CYBB and OE-CYBB+Erastin groups, indicating weakened capacity of iron storage and a relative increase in free iron pools. This was consistent with the rise in Fe^2+^. These results suggested that the overexpression of CYBB could induce ferroptosis in macrophages. The activation of ferroptosis was featured by a marked rise in intracellular Fe^2+^ and ROS, coupled with the transcriptional patterns of up-regulated ACSL4 and down-regulated SLC7A11, GPX4, and FTH1. In addition, lipid peroxidation was further evaluated by measuring malondialdehyde (MDA). Consistent with enhanced ferroptotic stress, CYBB overexpression significantly increased MDA levels compared with the control/OE-NC group, and Erastin stimulation further amplified MDA accumulation in OE-CYBB cells (**Figure 8A**). To corroborate the transcriptional findings at the protein level, western blotting demonstrated that CYBB overexpression led to a reduction in the anti-ferroptotic proteins GPX4 and SLC7A11, which was further decreased upon Erastin exposure (**Figures 8C, D**). Transmission electron microscopy further revealed canonical ferroptosis-associated mitochondrial morphology after erastin exposure, including mitochondrial shrinkage, increased membrane density, and loss of cristae (**Figure 8G**). CYBB overexpression alone elicited similar ultrastructural alterations, and co-treatment with erastin produced the most severe mitochondrial damage. These findings provided morphological support that CYBB primes macrophages for ferroptosis and amplified erastin-triggered ferroptotic stress. Collectively, these results provided convergent evidence that CYBB overexpression aggravated ferroptosis-related oxidative damage and compromised the GPX4–SLC7A11 antioxidant defense axis in macrophages.

### 3.8 CYBB knockdown attenuates iron/ROS accumulation and partially reverses

**Figure 9:**
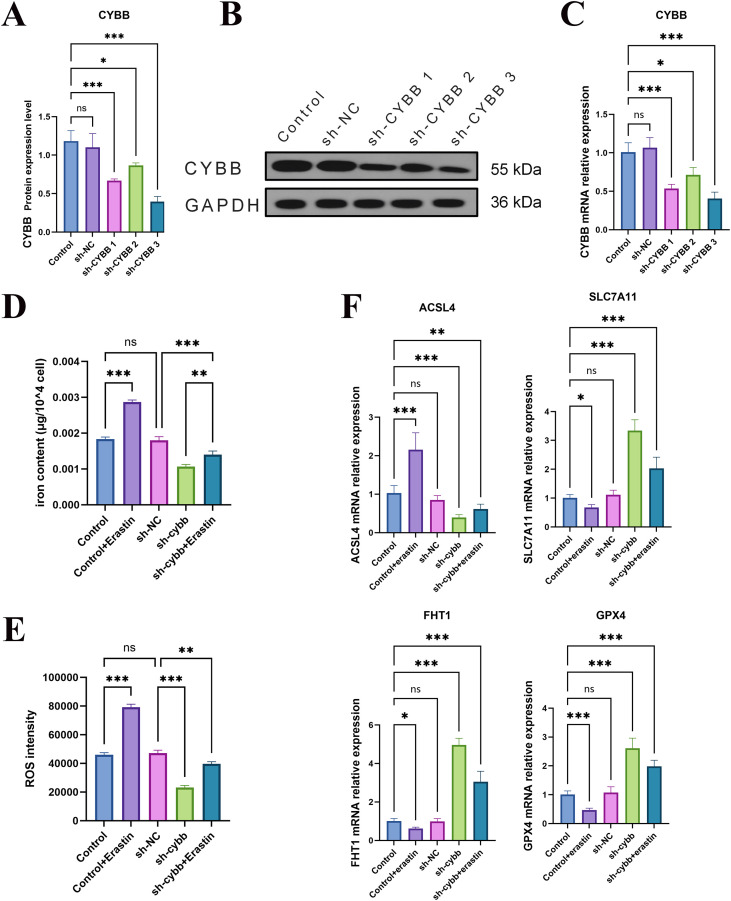
**(A)** Quantification of CYBB protein levels after transfection with sh-NC or three independent shRNAs targeting CYBB (sh-CYBB1/2/3). **(B)** Representative Western blot images showing CYBB protein expression (GAPDH as loading control). **(C)** qPCR validation of CYBB mRNA knockdown efficiency in sh-CYBB1/2/3. **(D)** Intracellular ferrous iron (Fe^2+^) content in Control, Control+Erastin, sh-NC, sh-CYBB, and sh-CYBB+Erastin groups. **(E)** Intracellular ROS intensity in the indicated groups. **(F)** qPCR analysis of ferroptosis-associated genes (SLC7A11, ACSL4, FTH1, and GPX4) under the indicated treatments. Data are presented as mean ± SD from n = 3 (for qPCR/Fe^2+^/ROS). Statistical significance was evaluated using one-way ANOVA followed by Tukey’s multiple-comparisons test. ns, not significant; P < 0.05, P < 0.01, P < 0.001, P < 0.0001. These symbols indicate statistical significance levels: *represents p<0.05; ** represents p<0.01; *** represents p<0.001.

To confirm CYBB silencing, we quantified CYBB expression at both the mRNA and protein levels using three independent shRNAs. Compared with the Control and sh-NC groups, CYBB knockdown markedly reduced CYBB expression, with sh-CYBB3 showing the highest silencing efficiency (**Figures 9A–C**). Functionally, Erastin stimulation significantly increased intracellular ferrous iron (Fe^2+^) content and ROS intensity in control cells, whereas CYBB knockdown substantially attenuated Fe^2+^ accumulation and ROS generation relative to sh-NC under the same treatment conditions (**Figures 9D, E**). Notably, although Erastin partially restored iron levels in CYBB-silenced cells, Fe^2+^ content remained significantly lower than that observed in Erastin-treated control cells (**Figure 9D**), indicating that CYBB contributed to Erastin-driven redox/iron stress in macrophages.

Consistent with these phenotypic changes, qPCR profiling of ferroptosis-related molecules demonstrated that CYBB knockdown shifted the transcriptional program toward a less ferroptosis-prone state. Specifically, compared with sh-NC, CYBB silencing alleviated the Erastin-associated suppression of antioxidant defense components (SLC7A11 and GPX4) and increased the expression of the iron-storage gene FTH1, while dampening the pro-ferroptotic marker ACSL4 (**Figure 9F**). Importantly, these transcriptional trends were further supported at the protein level: CYBB knockdown partially restored GPX4 and SLC7A11 protein abundance under oxidative stress conditions (**Figures 8E, F**), reinforcing a functional rescue of the GPX4–SLC7A11 axis. Moreover, lipid peroxidation assessed by MDA measurement was reduced following CYBB knockdown under comparable treatment conditions (**Figure 8B**), further indicating that CYBB depletion mitigated ferroptosis-associated oxidative damage. Consistently, TEM analysis showed that CYBB knockdown preserved mitochondrial integrity and attenuated ferroptosis-like ultrastructural injury under erastin challenge (**Figure 8G**). The reduced mitochondrial shrinkage and cristae disruption in sh-CYBB+erastin cells corroborated that CYBB was a key upstream determinant of macrophage ferroptosis susceptibility.

Together, these results demonstrated that CYBB was a key determinant of iron/ROS homeostasis and ferroptosis vulnerability in macrophages, and that CYBB knockdown dampened Erastin-induced ferroptotic responses by restraining Fe^2+^/ROS accumulation while partially restoring antioxidant defense.

## 4 Discussion

The characteristic pathological features of UC are inflammation initiating from the rectum and extending continuously proximally along the colon, primarily affecting the mucosa and submucosa with a superficial distribution. Recently, the function of immune cells in the imbalance of intestinal microenvironment has garnered widespread attention. Specifically, the dynamic balance of macrophages between sustaining inflammatory responses and promoting tissue repair has been increasingly explored. Through multilayer integration of scRNA-seq and Bulk RNA sequencing data, this study systematically delineated the single-cell transcriptomic landscape of macrophages in normal and UC tissues. Utilizing hdWGCNA and ML approaches, four hub genes (HIF1A, S100A8, CYBB, GLS) strongly linked to ferroptosis-related processes were ascertained. Focusing on CYBB, we further validated its functional link to the accumulation of ROS, disruption of iron homeostasis, and ferroptosis at both cellular and animal levels. Subsequently, four-gene tissue-based signature and nomogram were developed and validated for molecular stratification. Given that it is derived from colonic tissue transcriptomes, this signature is positioned as an adjunct/research-oriented tool rather than a direct replacement for routine UC diagnosis, and future studies will test its transferability to blood/plasma/fecal assays and prospectively validate it in independent cohorts. Notably, this research detected that ferroptosis represents a pivotal axis of immunometabolic dysregulation in macrophages within UC. However, macrophage activation in human UC mucosa is not adequately captured by a strict M1/M2 dichotomy. Accordingly, although we report CIBERSORT-estimated macrophage fractions using the LM22-derived M0/M1/M2 categories (**Figure 5B**), these bulk deconvolution labels represent coarse transcriptional surrogates and may not fully capture the spectrum of *in vivo* macrophage states. We did not impose a strict M1/M2 binary labeling for macrophages in the human UC mucosa. In inflamed intestinal tissue, macrophage activation is better described as a continuum shaped by microbial, cytokine, hypoxic, and tissue-remodeling cues, and canonical binary markers may be low or variable in human scRNA-seq data. Therefore, M1/M2 dichotomization may introduce annotation bias and oversimplify the underlying biology. In this study, we focused on contrasting disease-associated versus control macrophages (UC-M vs N-M, defined by donor disease status) and on network-level programs characterized by pseudotime and hdWGCNA. Future work will pursue finer-grained macrophage state mapping using integrative reference-based annotation and multi-modal validation. CYBB (NOX2) serves as an upstream promoter of this axis. The overexpression of CYBB exacerbates ferroptosis. Prior studies have exhibited that CYBB exerts dual effects in immune infiltration and ferroptosis. It is highly expressed in tumor-associated macrophages and engages in managing the sensitivity and polarization of ferroptosis, confirming CYBB as a critical nexus connecting immune responses and ferroptosis (20). These findings align with our observations in UC and further support a working model in which the CYBB–ROS–ferroptosis module is associated with polarization-related transcriptional programs and macrophage-mediated tissue responses. Notably, the causal relationship between ferroptotic signaling and macrophage polarization was not directly tested in this study; therefore, the polarization-related discussion should be interpreted as correlative.

*In vitro* knockdown of CYBB can upregulate anti-ferroptosis molecules like FTH1 and FSP1, promote M2-related markers, and inhibit M1 markers. This suggested a bidirectional modulatory effect of CYBB on ‘sensitivity of ferroptosis—balance of polarization’, which further reshapes the tumor immune microenvironment (20).

Studies from diverse disease models further demonstrate that CYBB is critical in the function of macrophages. At the developmental and homeostatic levels, the NOX complex is crucial for the differentiation of monocytes into macrophages and M2 polarization. Its deficiency reduces the production of ROS, impairs differentiation, and affects the generation of tumor-associated macrophage. This denotes that CYBB implicates in the differentiation of macrophages and regulates the transitions of functional state in macrophages (21). In injury and neurodegeneration models, NOX2 is highly expressed in M1-like microglia and macrophages. Genetic or pharmacological inhibition of NOX2 downregulates M1 markers and upregulates M2 markers, highlighting the ‘switch-like’ function of CYBB between M1 and M2 polarization (22). Regarding the regulation of inflammation, studies have exhibited that NOX2 participates in phagocytosis and bactericidal activity, modulates immune responses, and suppresses excessive inflammation through the Nrf2 pathway. Meanwhile, functional defects in NOX2 are associated with Crohn’s-like ileitis (23). A study more directly linked to colitis detected that blocking the interaction between eNAMPT and CYBB/TLR4 considerably alleviates DSS-induced colitis. This indicates that CYBB on the surface of macrophages serves as both a source of ROS and an integration node for inflammatory signals (24). Moreover, inhibiting CYBB or the activation of NADPH oxidase also exerts protective effects (25). Notably, the S100A8–NCF2/NOX2 axis promotes lipid peroxidation and downregulation of GPX4, suggesting that the ‘inflammatory signal-NOX2 activation-ferroptosis’ pathway is conserved across diverse tissues. This aligns with the mechanisms we uncovered in UC-M (26). In autoimmune diseases, deficiency of CYBB exacerbates lupus nephritis and activates the TLR7-NF-κB pathway, indicating that CYBB exerts protective effects in suppressing excessive immune responses and preventing tissue damage (27, 28). Therefore, CYBB may have dual ‘pro-inflammatory’ and ‘anti-inflammatory’ properties within the immune system. This characteristic suggests that the function of CYBB depends on the dynamic balance of the microenvironment. Under chronic inflammatory conditions such as UC, persistent overexpression or aberrant activation of CYBB might act as a key factor in disrupting homeostasis and inducing ferroptosis.

Moreover, our integrative analyses and multi-level validation support a working model in which CYBB-driven redox/iron stress is coupled to ferroptosis vulnerability in UC macrophages, providing a rationale for CYBB-centered mechanistic interrogation and stratification-oriented biomarker development. This research has several limitations. First, though we consistently detected concordant trends across independent datasets and *in vivo* and *in vitro* experiments, the precise upstream/downstream signaling by which CYBB modulates ferroptosis vulnerability in macrophages requires further delineation and temporal resolution. Second, while we observed coordinated changes in redox/iron stress, lipid peroxidation, and ferroptosis-related markers, the causal linkage between ferroptotic signaling and macrophage polarization programs was not directly tested and should be interpreted as correlative. Third, the bidirectional regulatory relationships between macrophages and epithelial/stromal compartments, as well as the contribution of cell–cell communication to ferroptosis-associated macrophage states, warrant further investigation. Overall, our findings support a CYBB-linked redox/iron–ferroptosis module in UC macrophages and provide a framework for future mechanistic and stratification-oriented studies.

## 5 Conclusion

In conclusion, four hub genes (HIF1A, S100A8, CYBB, GLS) strongly linked to ferroptosis in UC were ascertained. Also, their pivotal roles in immunopathology within UC were systematically validated utilizing scRNA-seq data and ML-based models. Among these genes, CYBB (the core catalytic subunit of the NADPH oxidase complex) is significantly upregulated in macrophages and is associated with increased oxidative/iron stress and a ferroptosis-prone molecular phenotype, characterized by ROS accumulation and perturbed iron homeostasis. This research supports a CYBB–ROS–ferroptosis module as a key component of macrophage dysregulation in UC, suggesting the potential utility of a CYBB-centered molecular signature for UC stratification and hypothesis-driven therapeutic prioritization.

## Data Availability

The original contributions presented in the study are included in the article/**Supplementary Material**. Further inquiries can be directed to the corresponding authors.

## Supplementary material

Supplementary Table 1 Marker genes used for annotation of major cell clusters.

Supplementary Table 2 List of 835 ferroptosis-related genes (FRGs) collected for this study.

Supplementary Table 3 Primer sequences used in this study.

Supplementary Table 4 Gene list displayed in **Figure 1H** heatmap (N-M vs UC-M macrophages).

Supplementary Table 5 Gene list displayed in **Figure 3D** heatmap.
