# Supplementary material for: Deciphering the macrophage ferroptosis regulatory network: construction of an ulcerative colitis diagnostic model and investigation of the immune microenvironment based on single-cell and transcriptomic data
Source: Front Immunol. 2026 Apr 22;17:1758082. doi: 10.3389/fimmu.2026.1758082 (PMC13143585; doi:10.3389/fimmu.2026.1758082)
Supplement: Supplementary Table 1 — Marker genes used for annotation of major cell clusters. [file Table1.docx]

| Cell | Features |
| --- | --- |
| T cell | “CD3D", "CD3E", "CD4", "CD8A" |
| B cell | "IGHG1","CD79B","CD79A","CD19" |
| Epithelial cell | "EPCAM","WFDC2","KRT8","CD24" |
| Endothetial cell | "ADGRL4","CLDN5","TCF4","PECAM1" |
| Fibroblast/Stromal cell | "COL6A2","MEG3","LUM","COL6A1" |
| Macrophage | "SOCS3","CSF1R","CD14","CD83" |
| NK cell | "KLRB1","KLRD1","NCR1","GNLY" |
| Plasma cell | "PRDM1","CD38","SDC1" |

**Table S1.** Marker Genes Used for Annotation of Major Cell Clusters
