# Supplementary material for: Deciphering the macrophage ferroptosis regulatory network: construction of an ulcerative colitis diagnostic model and investigation of the immune microenvironment based on single-cell and transcriptomic data
Source: Front Immunol. 2026 Apr 22;17:1758082. doi: 10.3389/fimmu.2026.1758082 (PMC13143585; doi:10.3389/fimmu.2026.1758082)
Supplement: Supplementary Table 3 — Primer sequences used in this study. [file Table3.doc]

| **Gene symbol** | **Forward Primer(5’→3’)** | **Reverse Primer(5’→3’)** |
| --- | --- | --- |
| β-actin | CACCATTGGCAATGAGCGGTTC | AGGTCTTTGCGGATGTCCACGT |
| GAPDH | GTCTCCTCTGACTTCAACAGCG | ACCACCCTGTTGCTGTAGCCAA |
| CYBB | CTCTGAACTTGGAGACAGGCAAA | CACAGCGTGATGACAACTCCAG |
| SLC7A11 | TCTCCAAAGGAGGTTACCTGC | AGACTCCCCTCAGTAAAGTGAC |
| GPX4 | GAGGCAAGACCGAAGTAAACTAC | CCGAACTGGTTACACGGGAA |
| ACSL4 | CATCCCTGGAGCAGATACTCT | TCACTTAGGATTTCCCTGGTCC |
| FTH1 | CCCCCATTTGTGTGACTTCAT | GCCCGAGGCTTAGCTTTCATT |

**Table S3.** Primer Sequences Used in This Study
